# Interconnecting Carbon Fibers with the *In-situ* Electrochemically Exfoliated Graphene as Advanced Binder-free Electrode Materials for Flexible Supercapacitor

**DOI:** 10.1038/srep11792

**Published:** 2015-07-07

**Authors:** Yuqin Zou, Shuangyin Wang

**Affiliations:** 1 State Key Laboratory of Chem/Bio-Sensing and Chemometrics, College of Chemistry and Chemical Engineering, Hunan University, Changsha, 410082, P. R. China; 2 School of Chemistry, The University of Manchester, Oxford Road, Greater Manchester, M13 9PL, United Kingdom

## Abstract

Flexible energy storage devices are highly demanded for various applications. Carbon cloth (CC) woven by carbon fibers (CFs) is typically used as electrode or current collector for flexible devices. The low surface area of CC and the presence of big gaps (ca. micro-size) between individual CFs lead to poor performance. Herein, we interconnect individual CFs through the *in-situ* exfoliated graphene with high surface area by the electrochemical intercalation method. The interconnected CFs are used as both current collector and electrode materials for flexible supercapacitors, in which the *in-situ* exfoliated graphene act as active materials and conductive “binders”. The *in-situ* electrochemical intercalation technique ensures the low contact resistance between electrode (graphene) and current collector (carbon cloth) with enhanced conductivity. The as-prepared electrode materials show significantly improved performance for flexible supercapacitors.

A fast-growing market for electronic devices and the development of hybrid electric vehicles result in an ever increasing demand for environmentally friendly and low-cost energy devices^1^,^2^,^3^,^4^,^5^,^6^. Supercapacitor, also known as ultracapacitor or electric double-layer capacitor, could store charges by separation of charges in a Helmholtz double layer at the interface between the surface of a conductive electrode and an electrolyte. Thus, due to their simple charge storage mechanism, supercapacitors are able to store and deliver energy at relatively high rates. Currently, carbon-based electrode materials ranging from activated carbon to carbon nanotubes and graphene are the most commonly utilized in supercapacitors because of their excellence physical and chemical properties^7^,^8^,^9^. In the development of supercapacitors, a proper control over the specific surface area and optimized contact between electrode materials and current collectors are crucial to ensure a good performance of the supercapacitor in terms of both power delivery rate and specific capacitance.

With rapidly growing demand for personal electronics with small, thin, light-weight, flexible, and even roll-up characteristics, more and more attentions have been devoted to the flexible energy storage systems including flexible supercapacitors for these electronic devices^10^. For a flexible supercapacitor, the vital component is a flexible electrode with favourable mechanical strength and large capacitance. It is still a challenging task to fabricate a supercapacitor electrode with the advanced characteristics of light-weight, flexibility, high conductivity and high surface area. In general, flexible electrodes could be prepared by fabricating free-standing film of the active materials, or by depositing active materials on flexible substrate^11^,^12^,^13^,^14^. Typically, carbon nanotube- or graphene-based composite thin films prepared by vacuum filtration have been well-developed for flexible supercapacitors^15^. However, the free-standing thin films developed this way showed poor mechanical strength, which would hinder the potential application of supercapacitors in some strict environment. On the other hand, recently, researchers developed a variety of approaches to deposit/load active electrode materials onto the flexible current collectors. For examples, Shi *et al.*^5^ coated cotton paper with carbon nanotubes by immersing the paper into the carbon nanotube solutions. Cui *et al.*^16^ fabricated flexible supercapacitors by directly drawing graphite on cellulose paper. Previously, we also developed the electrophoresis deposition method to load porous graphene and porous graphene/carbon nanotubes hybrid onto carbon cloth as the electrode materials of flexible supercapacitors^11^,^12^. However, supported electrode materials fabricated this way usually have high contact resistance between electrode materials and current collectors (substrates), which would hinder the performance of the flexible supercapacitors.

Carbon cloth, which is mechanically flexible carbon fibers woven in form of cloth, could be attractive electrode for flexible supercapacitor because of their good electrical conductivity, chemical stability, flexibility and high porosity. However, carbon cloth only shows a very low surface area due to the large size of carbon fibers (around 10 μm). During the production of carbon cloth, inevitably, big gaps (*ca.* micro-size) between individual carbon fibers are generated, as observed by the scanning electron microscopy (SEM) images (Figure S1, Supporting Information) which would significantly reduce the area-normalized capacitance when used as the electrode of supercapacitors. In this work, for the first time, we utilized electrochemical cation intercalation method to *in-situ* exfoliate graphene from carbon fibers of carbon cloth. The as-exfoliated graphene interconnected individual carbon fibers, which showed significantly increased surface area. The electrochemical cation intercalation method used here is a non-oxidative production route to few layer graphene^7^, which avoid the further reduction of oxidized graphene for use in supercapacitors. The interconnecting of carbon fibers by graphene (as a conductive “binder”) would enhance the conductivity of the composites. The interconnected carbon fibers by graphene showed significantly improved specific capacitance as the binder-free electrode materials for flexible supercapacitors in terms of area-normalized capacitance.

## Results

**Figure 1:**
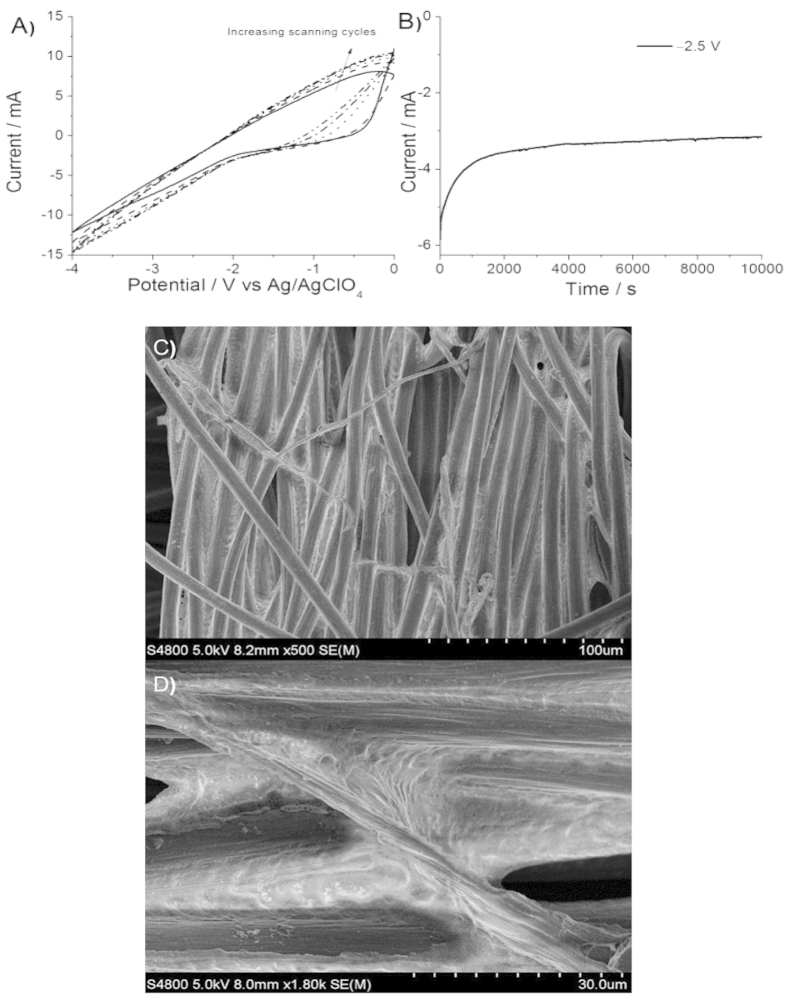
(**A**) The cyclic voltammetry curves of carbon cloth in 0. 1 M TMAClO4 in NMP recorded at a scan rate of 20 mV/s; (**B**) the chronoamperometric curve of carbon cloth for the mild exfoliation at the constant potential of −2.5 V; SEM images of interconnected carbon fibers by the *in-situ* electrochemically exfoliated graphene (Ex-CC) at low magnification (**C**) and high magnification (**D**).

The electrochemical cation intercalation of carbon cloth was conducted by using a three-electrode system with carbon cloth as working electrode, Pt mesh as counter electrode, Ag/AgClO_4_ as reference electrode, and tetramethylammonium perchlorate (TMAClO_4_) as electrolyte. We performed the cyclic voltammetry (CV) scanning to define a proper potential for the chronoamperometric (CA) mode to start the electrochemical cation intercalation process. As shown by the CV curves in Fig. 1A, scanning from 0 to −4 V resulted in a clear cathodic current (starting from around −2 V) associated with the intercalation of cations into the graphite of carbon fibers^17^,^18^. At more negative potential, the current is much higher, indicating obviously better intercalation efficiency^17^. Initially, we conducted the CA running at a constant most negative potential of −4 V for 10000 s. At this potential, graphene was successfully exfoliated from graphite of carbon fibers, but most of them were de-attached from carbon fibers into the electrolyte and participated, as shown by the digital photograph in Figure S2. For the final use in flexible supercapacitor, it is required to keep the exfoliated graphene in the matrix of carbon fibers. Therefore, the potential of −4 V is too negative and too strong for this purpose. Subsequently, a milder potential of −2.5 V was chosen for the CA running (Fig. 1B) to exfoliate graphene from carbon fibers. After the intercalation process of 10000 s, no apparent participates were observed, indicating that the as-exfoliated graphene was well reserved in the matrix of carbon fibers. The SEM images were collected for the exfoliated carbon cloth (denoted as Ex-CC) obtained at the potential of −2.5 V. Different from the pristine carbon cloth (SEM images shown in Figure S1) in which carbon fibers were individually distributed and showed big gaps of around 1–4 μm, the carbon fibers in Ex-CC were interconnected by the *in-situ* exfoliated graphene, as shown in Figs 1C,D. As observed in Figs 1C,D, it seems that the exfoliated graphene acted as conductive “binder” to interconnect the individual carbon fibers. The conductive “binder” (graphene) linked with carbon fibers could effectively enhance the conductivity of the composites due to the high conductivity of graphene. The transmission electron microscopy (TEM) and atomic force microscopy (AFM) images were used to identify the structural information of the as-obtained materials. The TEM and AFM images, as shown in Figure S3, show the typical graphene structure. The graphene–interconnected carbon fibers would contribute more available charging sites per a unit area due to the high surface area of graphene, thus leading to higher area-normalized capacitance when used in supercapacitors.

**Figure 2:**
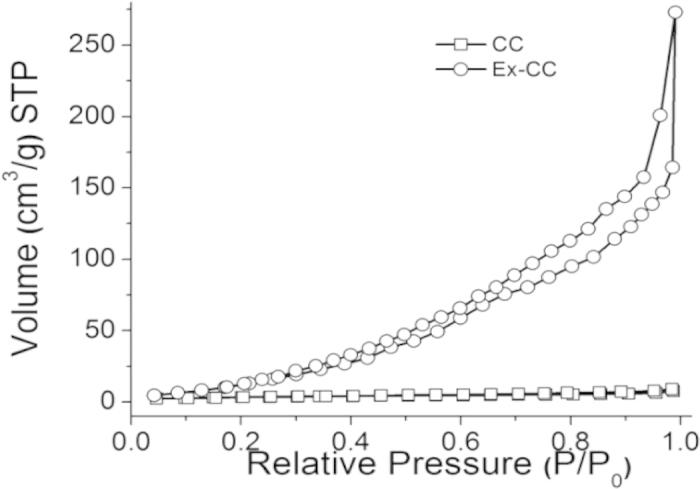
Nitrogen adsorption-desorption isotherm curves of Ex-CC and CC.

Based on the SEM observation, graphene was successfully exfoliated and acted as interlinkers to interconnect individual carbon fibers. It is well-known that graphene always has high surface area, therefore, it is expected that the interconnected carbon fibers by graphene, that is, Ex-CC would show much higher surface area than pristine CC. We performed the Brunauer Emmett Teller (BET) testing for Ex-CC as well as CC for comparison. Figure 2 showed the nitrogen adsorption-desorption isotherms of CC and Ex-CC. As can be seen, CC gave type I isotherms characterized by a plateau that is nearly horizontal to the P/P_0_ axis, indicating the microporous nature of of carbon fibers in CC^19^. For Ex-CC, the type IV isotherm with pronounced adsorption at low and medium relative pressures indicate the existence of a large number of mesopores and micropores created by the as-exfoliated graphene in Ex-CC. The hysteresis loop in the isotherms of Ex-CC indicates the Ex-CC is porous. The total pore volume of Ex-CC is 0.424 cm^3^/g, much higher than that of CC (0.011 cm^3^/g). After the electrochemical cation intercalation, Ex-CC exhibited much higher surface area (68.5 m^2^/g) than CC without intercalation (11.5 m^2^/g). The enhancement of the surface area by the *in-situ* interconnected graphene could be clearly found.

The electronic properties of Ex-CC and CC were investigated by Raman spectra. Raman spectroscopy is an excellent tool for investigating the electronic structure and defect concentration in graphene^20^,^21^,^22^,^23^. As can be seen from the Raman spectra in Figure S4, the *D* band and *G* band were located around 1330 and 1580 cm^−1^, respectively. It has been found that *G* band arises from the bonding stretching of sp^2^-bonded C-C pairs, while the *D* band is associated with the sp^3^ defect sites. In the Raman spectra of carbon-based materials, the ratio of I_D_/I_G_ was usually used as an indicator of the defects level. It can be seen from Figure S4 that Ex-CC showed slightly higher I_D_/I_G_ ratio than pristine CC. For Ex-CC, graphite in carbon fibers was exfoliated to graphene, resulting in more exposure of edge defects and thus higher D band intensity in the Raman spectrum.

**Figure 3:**
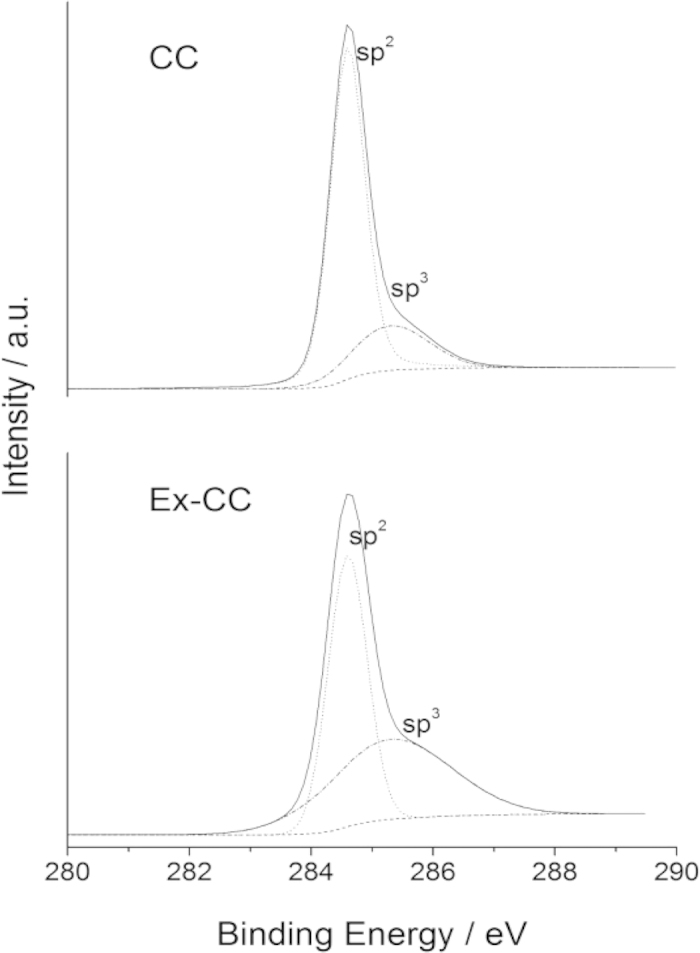
High resolution C1s XPS peak of CC and Ex-CC.

In order to monitor the change of the C bond configuration, fine-scan C1s spectra were collected from both CC and Ex-CC, as shown in Fig. 3. For CC as a typical carbon material, the C1s XPS peak could be fitted into two peaks, located around 284.6 and 285.3 eV, assigned to sp^2^ and sp^3^ C1s, respectively^24^. After the electrochemical intercalation, the sub-peak of sp^3^ C of Ex-CC increased relative to the pristine CC, indicating more edge defects exposed after exfoliation, in consistent with the observation of the Raman spectra as discussed above. It should be pointed out that no obvious oxygen-containing species were observed for Ex-CC, indicating the electrochemical cation intercalation is a non-oxidative route, which preserved the highly conductive properties of graphene.

**Figure 4:**
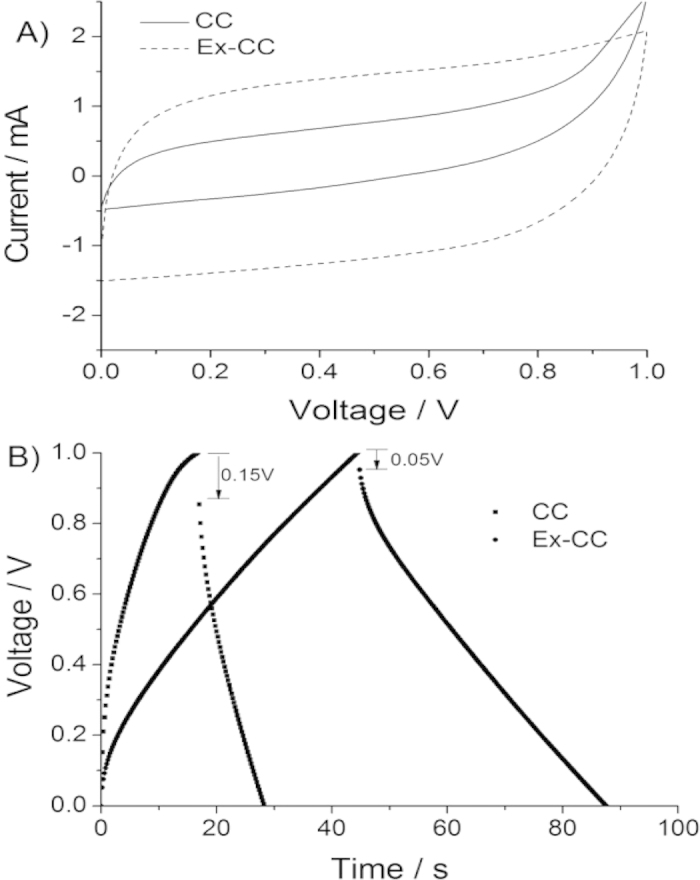
Cyclic voltammetry at the scan rate of 10 mV/s (A) and charge/discharge at the current load of 3 mA (B) curves of flexible supercapacitors based on the CC and Ex-CC electrode materials with 1 M H_2_SO_4_ as the electrolyte.

Based on the above physical and chemical characterizations, it is expected that Ex-CC would have attractive electrochemical performance in supercapacitors. Since the Ex-CC with high surface area originates from CC and preserves the well-defined cloth structure with good mechanical strength, Ex-CC could be an excellent candidate as an advanced binder-free electrode for flexible supercapacitors. The symmetric flexible supercapacitors were constructed using two Ex-CC samples as both positive and negative electrodes, Whatman filter membrane as the separator, and 1.0 M H_2_SO_4_ solution as the electrolyte. For comparison, pristine CCs were also assembled to a symmetric flexible supercapacitor. The area of the devices is 2 × 2 cm^2^. CV measurements were first carried out to observe the electrochemical behaviour of Ex-CC and CC-based flexible supercapacitors. Figure 4A shows the CV curves of the two flexible supercapacitors using Ex-CC and CC as electrodes, from which the remarkable difference in the electrochemical behaviour and properties between the two electrodes could be easily recognized. The CV curve at the Ex-CC electrode is close to the ideal rectangular shape, indicating smaller internal resistance in the electrode, while CC shows a very poor rectangular shape. Although carbon fiber is a highly conductive substrate, carbon fibers in CC have poor affinity with electrolytes due to the inert surface. After the electrochemical cation intercalation, graphite in the fibers expanded to graphene with more edge defects exposed, which may increase the affinity with electrolyte through the capillary interaction. Thus, Ex-CC is more electrochemically affinitive toward electrolyte. On the other hand, for Ex-CC, the interconnecting of carbon fibers by the *in-situ* exfoliated graphene would enhance the conductivity of the composites for use as electrode materials. From the CV curves, it could be observed that Ex-CC showed much higher current than CC, which could be attributed to the unique structure of the interconnected carbon fibers through the *in-situ* exfoliated graphene.

In order to further investigate the performance of the two electrodes, galvanostatic charge-discharge (CD) experiments were carried out with the voltage windows the same as for the above CV analysis. As shown in the CD curves (Fig. 4B), the discharging time of the Ex-CC was significantly longer than that of CC, indicating that the Ex-CC offers a much larger capacitance, which agrees well with those obtained from the CV testing. Moreover, for the galvanostatic charge-discharge, the *IR* drop of the Ex-CC is much smaller than CC. The *IR* drop is caused by the equivalent series resistance (ESR), which includes electrode resistance and electrolyte resistance. The smaller *IR* drop on Ex-CC is attributed to the existence of various pore structure and high conductivity of the *in-situ* exfoliated graphene. The specific capacitance in this work was calculated from the CD curves. In addition, the *in-situ* exfoliated graphene filled the gaps between individual carbon fibers in Ex-CC samples; therefore, it is interesting to investigate the contribution of the *in-situ* exfoliated graphene to the area-normalized capacitance. So, in this work, the specific capacitances were described with “*mF cm*^−2^”. According to the calculation, the area-normalized capacitance of Ex-CC at the discharge current of 3 mA is 64.5 mF cm^−2^, much high than that of CC (17.1 mF cm^−2^). The higher area-normalized capacitance of Ex-CC may be attributed to the presence of the *in-situ* exfoliated graphene with high surface area and high conductivity. Furthermore, we investigated the durability of the Ex-CC electrode using continuous charge/discharge cycles at a constant current load of 5 mA. Figure S5 shows the specific retention of Ex-CC as a function of cycle number. It can be clearly seen that Ex-CC shows a good cycling behaviour in supercapacitors and the Ex-CC remains stable under the electrochemical operation conditions.

Finally, we examined the electrochemical performance of the as-fabricated flexible supercapacitor based on Ex-CC as the electrode materials under bending conditions. Figure S6 shows the CV curves of the Ex-CC-based flexible supercapacitor device before and after bending, and it could be observed that the electrochemical performance of the device does not significantly change under the bending conditions, indicating the as-fabricated supercapacitors are highly flexible^25^.

## Discussion

Based on the above analysis, it could be found the individual carbon fiber in the carbon cloth could be efficiently interconnected by the electrochemically exfoliated graphene. The morphology characterizations demonstrate the ideal structure for the potential applications in supercapacitor due to more exposed active sites for charge storage. On the other hand, the enhanced capacitance as demonstrated by the electrochemical testing may also be attributed to the increased conductivity of the composite. The exfoliated graphene could significantly decrease the contact resistance between individual carbon fibers by linking each together.

In summary, we successfully interconnected carbon fibers of CC with graphene through the *in-situ* electrochemical exfoliation method. The electrochemical exfoliation leads to generation of graphene without de-attachment from carbon fiber under mild exfoliation conditions and preserves the high conductivity of graphene without the formation of oxygen-containing species. The exfoliated graphene, which remains in the matrix of carobn fibers, acted as conductive “binders” to enhance the conductivity and to increase the surface area of the composites^26^,^27^,^28^. The as-obtained Ex-CC were used as advanced binder-free electrode materials for flexible supercapacitor, which shows significantly enhanced supercapacitor performance compared to CC in terms of the area-normalized capacitance due to the high surface area and high conductivity of graphene in the cloth matrix. We also demonstrated that the Ex-CC based supercapacitors are very flexible, showing potential applications in the field of flexible electronic devices. Therefore, the strategy developed in this work has a significant impact on the development of the electrode materials for flexible energy storage system.

## Methods

### Preparation of Ex-CC

The electrochemical exfoliation of carbon cloth was conducted in a three-electrode system with carbon cloth as working electrode, Pt mesh as counter electrode and Ag/AgClO_4_ as reference electrode in 0.1 M TMAClO_4_ in NMP as electrolyte. First of all, Voltammetry was performed as a scan rate of 20 mV/s. The potentials observed in the cyclic voltammetry (CV) were used to define the potential set in the chronoamperometric mode to control the mild intercalation process. Subsequently, −2.5 V was chosen for the chronoamperometric mode to realize the mild electrochemical exfoliation for 10000 s. Following the electrochemical exfoliation, the as-obtained Ex-CC was washed with acetone thoroughly.

### Assembly of flexible supercapacitors

To construct Ex-CC based flexible supercapacitor device, two pieces of Ex-CC electrodes with the size of 2 × 2 cm^2^ were used as the positive electrode and negative electrode, respectively. Whatman membrane was used as the separator and 1 M H_2_SO_4_ in aqueous was used as electrolyte. For comparison, CC based supercapacitors were also assembled in the similar way.

### Electrochemical measurements

Cyclic voltammetry and galvanostatic charge/discharge tests of assembled two-electrode supercapacitors were carried out using an Autolab potentiostat/galvanostat. CV measurements were conducted in the applied voltage window of 0–1 V. Galvanostatic charge/discharge tests were operated under a constant charge/discharge current of 3 mA within an applied voltage window range from 0 to 1 V. The specific capacitance was calculated from the galvanostatic charge/discharge curves normalized by the surface area of the devices (4 cm^2^).

## Additional Information

**How to cite this article**: Zou, Y. and Wang, S. Interconnecting Carbon Fibers with the *In-situ* Electrochemically Exfoliated Graphene as Advanced Binder-free Electrode Materials for Flexible Supercapacitor. *Sci. Rep.* **5**, 11792; doi: 10.1038/srep11792 (2015).

## Supplementary Material

Supplementary Information
