# Supplementary material for: Interconnecting Carbon Fibers with the In-situ Electrochemically Exfoliated Graphene as Advanced Binder-free Electrode Materials for Flexible Supercapacitor
Source: Sci Rep. 2015 Jul 7;5:11792. doi: 10.1038/srep11792 (PMC4493559; doi:10.1038/srep11792)
Supplement: Supplementary Information [file srep11792-s1.doc]

**Supporting Information**

Interconnecting Carbon Fibers with the In-situ Electrochemically Exfoliated Graphene as Advanced Binder-free Electrode Materials for Flexible Supercapacitor

*Yuqin Zou, ^a,b^ & Shuangyin Wang*^a,b^*

**Supplementary Results:**


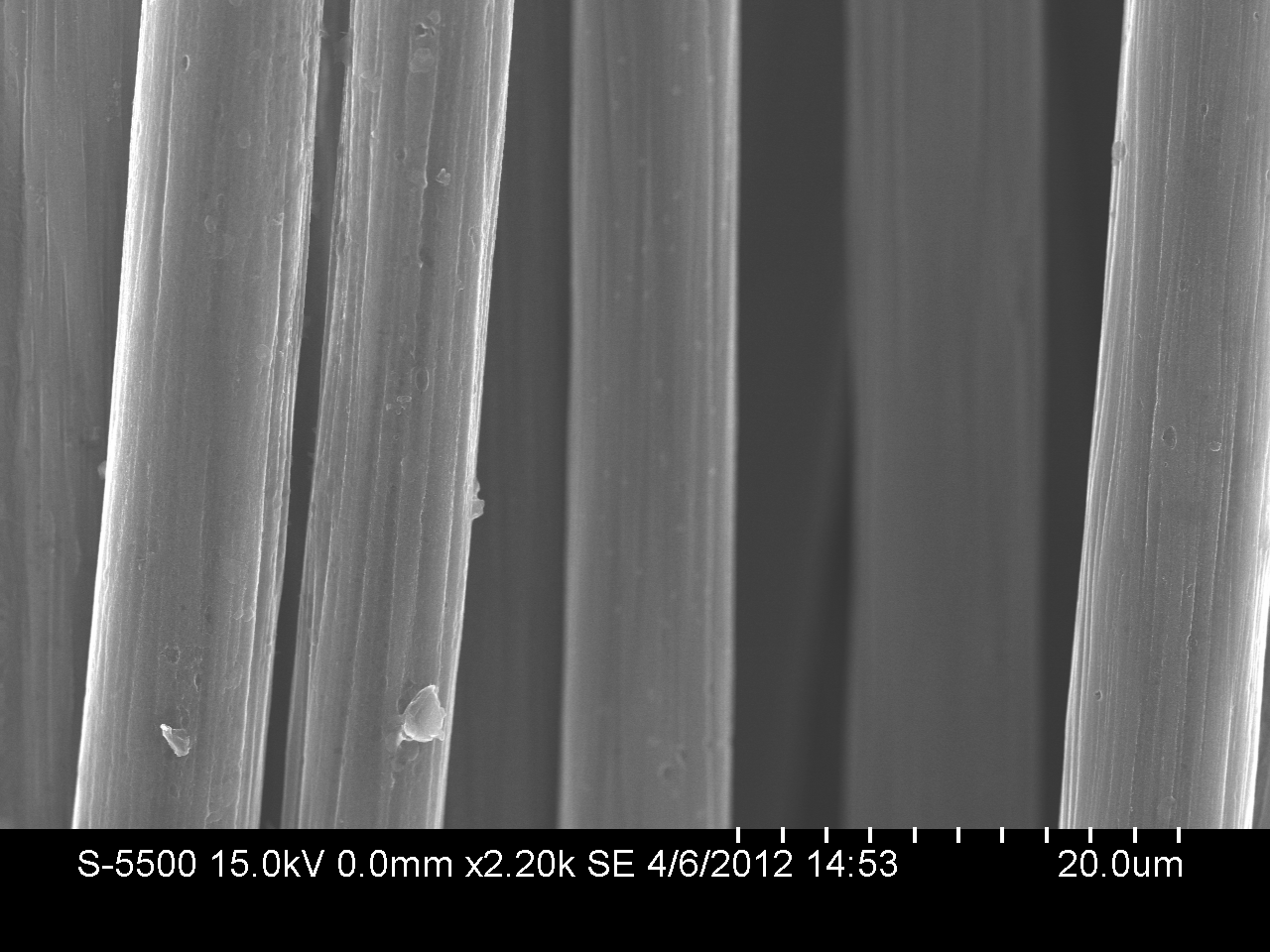

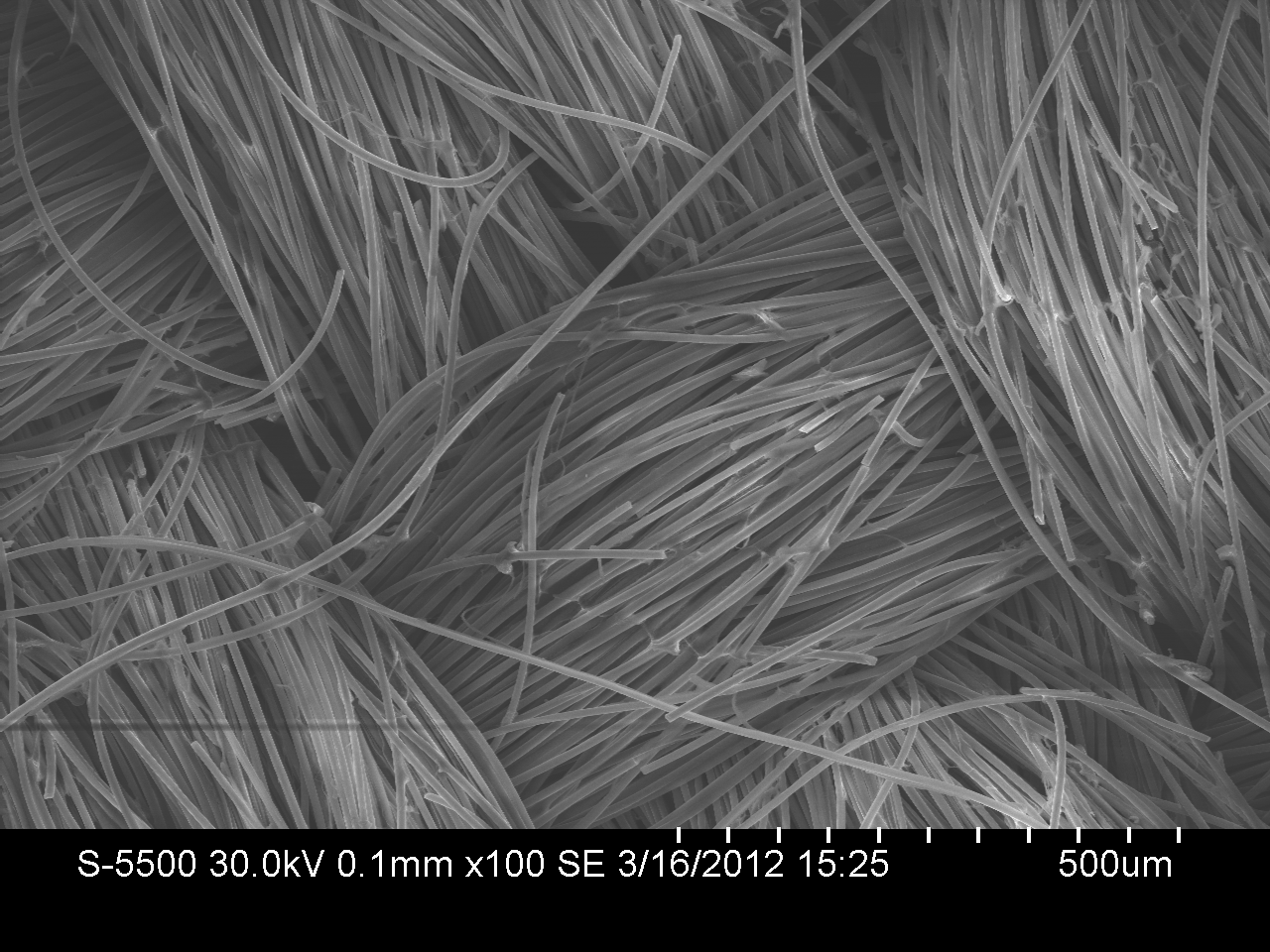


**500 µm**

**Figure S1**. SEM images of pristine carbon cloth.


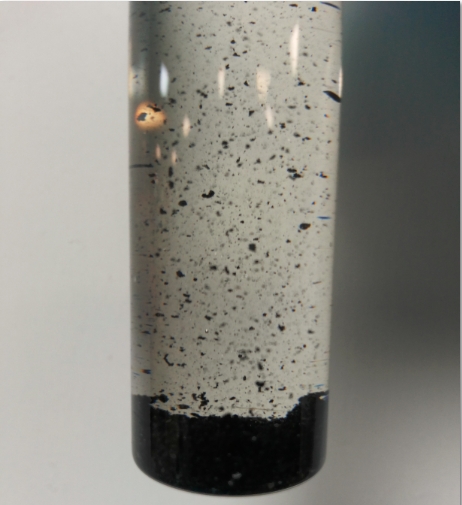


**Figure S2**. Photographene of the participates in the electrolyte after the electrochemical exfoliation of CC at a constant potential of -4 V.


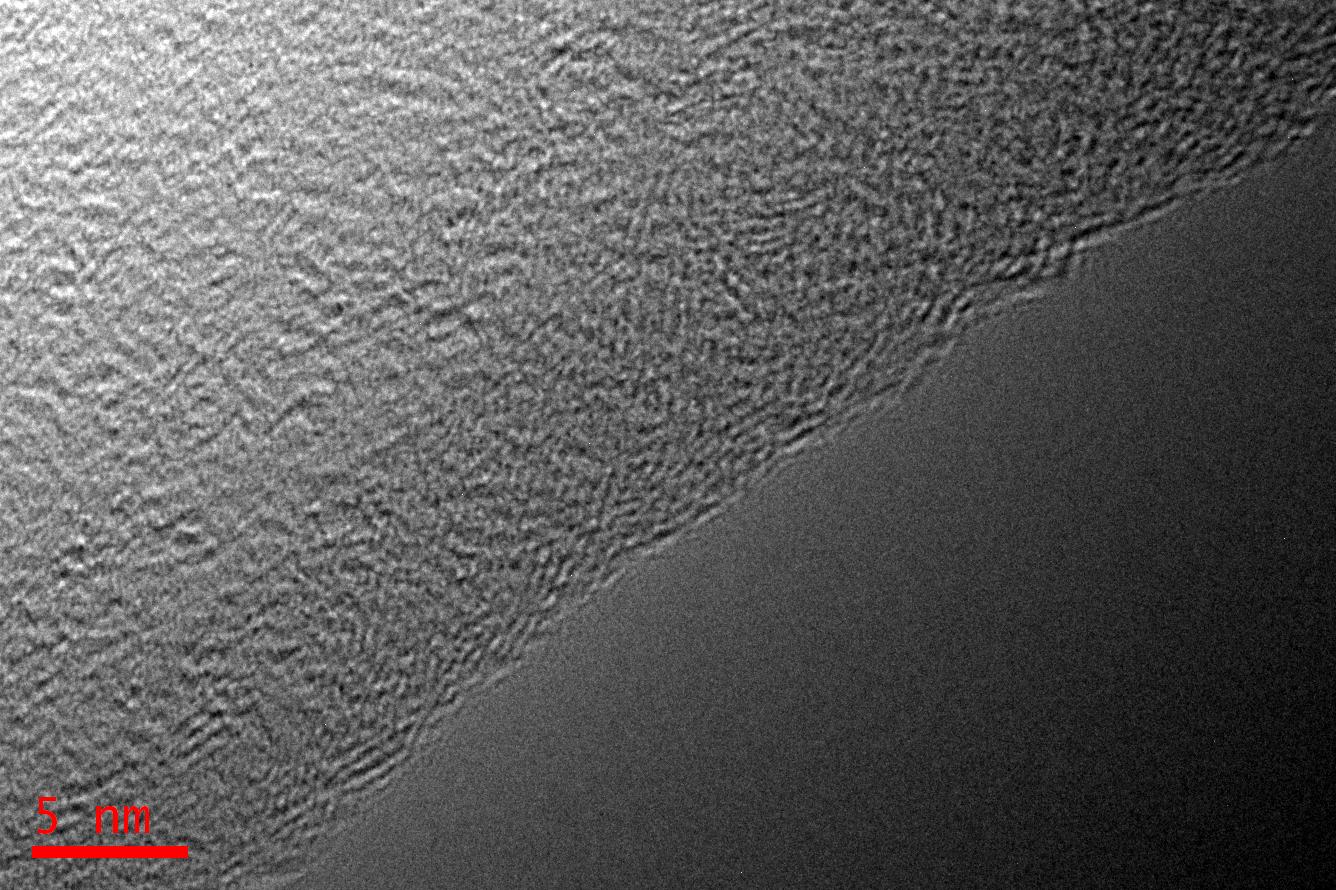

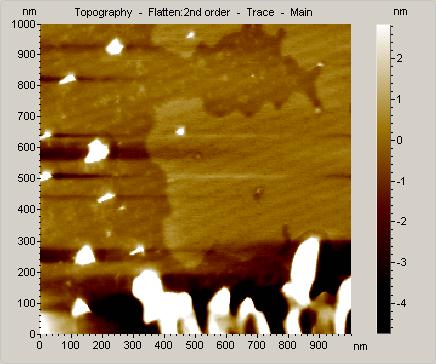


**Figure S3.** TEM and AFM image of the graphene from Ex-CC.


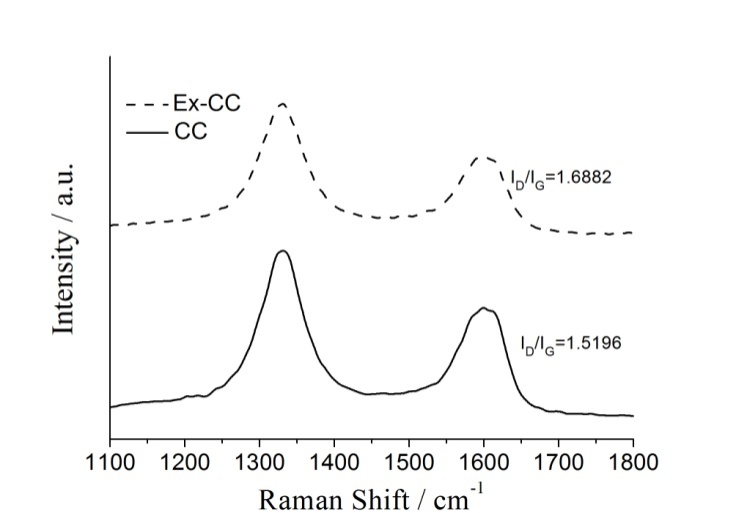


**Figure S4.** Raman shift of Ex-CC and CC.


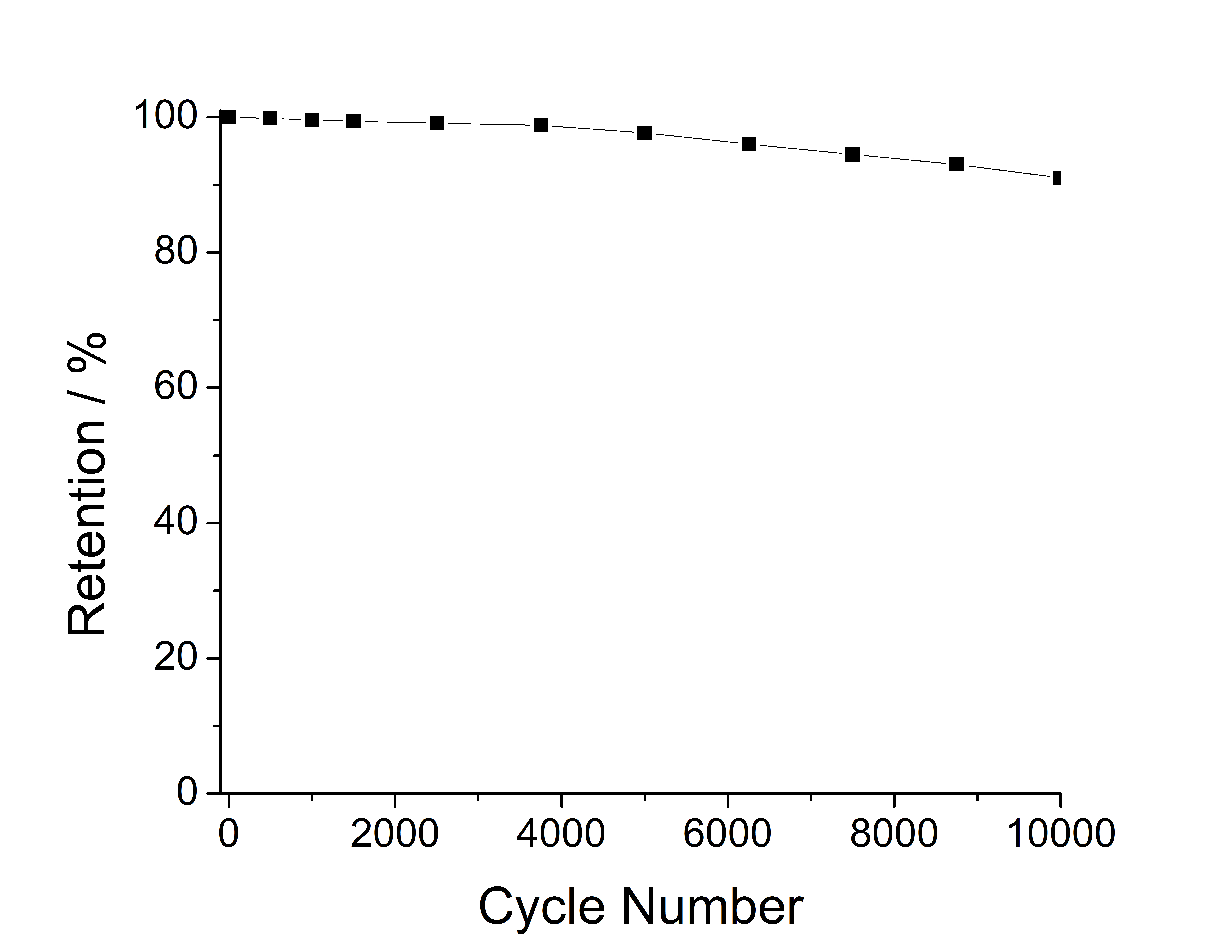


**Figure S5** Durability testing of Ex-CC based flexible supercapacitor.


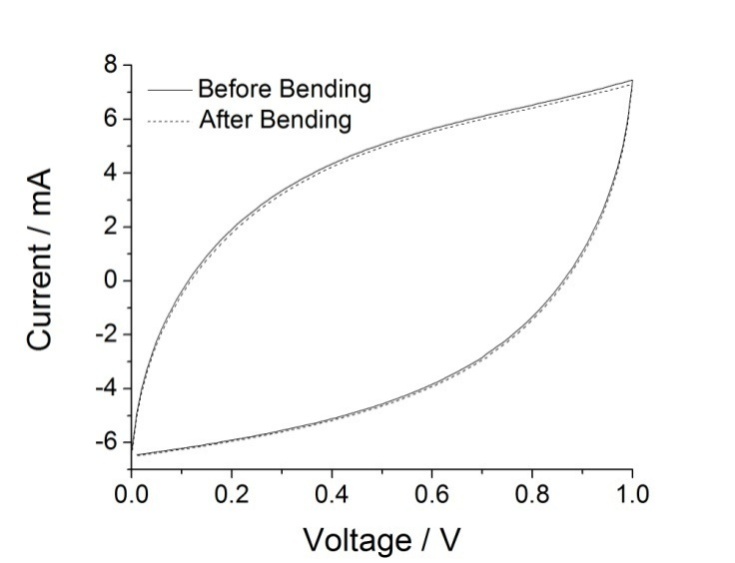


**Figure S6**. Bending testing of the as-assembled flexible supercapacitors based on Ex-CC at the scan rate of 50 mV/s.
